# Secondary hyperparathyroidism in chronic kidney disease: A narrative review focus on therapeutic strategy

**DOI:** 10.1016/j.clinme.2024.100238

**Published:** 2024-08-30

**Authors:** Shin-Hwa Tsai, Wei-Chih Kan, Rong-Na Jhen, Yu-Ming Chang, Jsun-Liang Kao, Hsien-Yung Lai, Hung-Hsiang Liou, Chih-Chung Shiao

**Affiliations:** a Department of Internal Medicine, National Taiwan University Hospital, No.7, Zhongshan S. Rd., Zhongzheng Dist., Taipei City, 100225, Taiwan, ROC; b Department of Nephrology, Department of Internal Medicine, Chi Mei Medical Center, No.901, Zhonghua Rd., Yongkang Dist., Tainan City, 71004, Taiwan, ROC; c Department of Medical Laboratory Science and Biotechnology, Chung Hwa University of Medical Technology, No.89, Wenhua 1st St., Rende Dist., Tainan City, 71703, Taiwan, ROC; d Division of Nephrology, Department of Internal Medicine, Camillian Saint Mary's Hospital Luodong, No. 160, Zhongzheng S. Rd., Luodong Township, 265, Yilan County, Taiwan, ROC; e Department of Anesthesiology, Da Chien General. Hospital, No. 36 Gongjing Rd., Miaoli City, Miaoli County, 360012, Taiwan, ROC; f Division of Nephrology, Department of Internal Medicine, Hsin-Jen Hospital, No. 387 Chong-Cheng Rd., Xinzhuang District, 242009, New Taipei City, Taiwan, ROC

**Keywords:** Calcimimetics, Chronic kidney disease, Parathyroidectomy, Parathyroid hormone, Secondary hyperparathyroidism, Vitamin D receptor activator

## Abstract

Chronic kidney disease (CKD) affects over 10% of the global population. One crucial complication of CKD is secondary hyperparathyroidism (SHPT), marked by elevated parathyroid hormone levels due to hyperphosphataemia, hypocalcaemia, and low active vitamin D from impaired renal function. SHPT increases risks of bone deformities, vascular calcification, cardiovascular events and mortality. This review examines SHPT treatment strategies in patients with CKD. First-line treatments include phosphate binders, vitamin D receptor activators and calcimimetics. When these fail, invasive options like parathyroidectomy (PTX) and thermal ablation are considered. PTX effectively reduces symptoms and improves radiological outcomes, outperforming medical treatment alone in reducing cardiovascular risk and mortality. Thermal ablation techniques, such as microwave, radiofrequency, laser or high-intensity focused ultrasound, offer less invasive alternatives with promising results. Future research should explore the molecular mechanisms of parathyroid gland hyperplasia and evaluate various treatments’ impacts.

## Introduction

Chronic kidney disease (CKD) is a disease with an increasing incidence and prevalence that affects over 10% of the general population.^1^ As kidney function declines, a complex disarrangement affecting bone, mineral metabolism and cardiovascular systems, named CKD–mineral bone disorder (CKD-MBD) occurs.^2^ Subsequently, the progressive decreased calcium levels or increased phosphate levels in advanced CKD stages induce overactive parathyroid glands, called secondary hyperparathyroidism (SHPT), that is typically characterised by hypocalcaemia, hyperphosphataemia, elevated fibroblast growth factor-23 (FGF-23), reduced 1,25-dihydroxy vitamin D_3_ [1,25(OH)_2_D_3_] and high parathyroid hormone (PTH) levels.^3^

Abnormalities in bone metabolism and heterotopic calcifications are common in SHPT, with radiographic features including subperiosteal bone resorption, Rugger jersey spine sign, brown tumours, pathological fractures, and deformities in severe cases.^4^ Besides bone and joint disorders, SHPT is also associated with symptoms like pruritis and muscle soreness,^5^ and some long-term consequences include renal osteodystrophy, heightened fracture risk, osteoporosis, vascular calcification, immune dysfunction, and renal hyporesponsive anaemia, imposing a significant economic burden.^3^^,^^6^ Further, the high PTH levels in patients with SHPT correlate with increased all-cause and cardiovascular mortality.^7^

For patients with CKD equal to or more advanced than stage 3, guidelines suggest regular monitoring of calcium, phosphate and PTH levels with intervals varied by stages of CKD^8^, ^9^, ^10^ (Table 1). As to the recommended PTH targets for CKD-MBD patients, the National Kidney Foundation's Kidney Disease Outcomes Quality Initiative (KDOQI) guideline published in 2003 suggests target ranges of PTH levels that are different in different stages of CKD.^8^ The 2009 Kidney Disease Improving Global Outcomes (KDIGO) guideline suggests that PTH levels in dialysis patients should be maintained at two to nine times the upper reference limit (URL), while suggestions for CKD patients without dialysis are lacking.^9^ The goals for PTH levels are agreed upon in the 2017 KDIGO guidelines for CKD-MBD^10^ and are commonly adopted in Asian countries.^11^ (Table 1)

**Table 1:** Target ranges of relevant biochemistries of SHPT by stages of CKD.

	**Stage 3** eGFR = 30–59 mL/min/1.73 m^2^	**Stage 4** eGFR = 15–29 mL/min/1.73 m^2^	**Stage 5** eGFR <15 mL/min/1.73 m^2^	
			without dialysis	with dialysis

**Target ranges of serum phosphate**				

KDOQI 2003^8^	2.7–4.6 mg/dL [0.87–1.48 mmol/L]		3.5–5.5 mg/dL [1.12–1.76 mmol/L]	

KDIGO 2009^9^ & 2017^10^	Maintain within normal range or lower toward the normal range 2.7–4.6 mg/dL [0.87–1.48 mmol/l]			

JSDT 2013^12^	Not mentioned		3.5–6.0 mg/dL [1.12–1.92 mmol/L]	

**Target ranges of corrected calcium**				

KDOQI 2003^8^	Within normal range: 8.4–10.5 mg/dL [2.1–2.6 mmol/L]		Within the normal range, prefer toward the lower end: 8.4 mg/dL [2.1 mmol/L]	

KDIGO 2009^9^ & 2017^10^	Maintain within normal range 8.4–10.5 mg/dL [2.1–2.6 mmol/L]			

JSDT 2013^12^	Not mentioned		8.4–10.0 mg/dL [2.1–2.5 mmol/L]	

**Target ranges of PTH**				

KDOQI 2003^8^	35–70 pg/mL [3.9–7.7 pmol/L]	70–110 pg/mL [7.7–12.1 pmol/L]	150–300 pg/mL [16.5–33.0 pmol/L]	

KDIGO 2009^9^ & 2017^10^	Not known, suggest evaluating for modifiable factors for those above URL (*65 pg/mL [7.2 pmol/L])			Within 2–9x URL (*130–585 pg/mL [14.3–64.4 pmol/L])

JSDT 2012^12^	Not mentioned			60–240 pg/mL [6.6–26.4 pmol/L]

*Abbreviation:* eGFR, estimated glomerular filtration rate; JSDT, Japanese Society for Dialysis Therapy; KDIGO, Kidney Disease Improving Global Outcomes; KDOQI, Kidney Disease Outcomes Quality Initiative; PTH, parathyroid hormone; URL, upper reference limit.

Note: *calculated using the reference ranges of serum intact-PTH (14–65 pg/mL [1.5–7.2 pmol/L]).

SHPT is diagnosed by biochemical abnormalities characterised by elevated levels of PTH, often accompanied by hyperphosphataemia, hypocalcaemia and vitamin D deficiency. The initial guidelines for diagnosing SHPT were established by the KDOQI in 2003. These recommendations were provided for patients with CKD stages 3 to 5, emphasising the importance of monitoring serum levels of calcium, phosphate and PTH.^8^ The KDOQI 2003 guideline recommends various target PTH levels in different CKD stages, with a target PTH level of 150–300 pg/mL (16.5–33.0 pmol/L) for patients with CKD stage 5 irrespective of undergoing dialysis.^8^ However, subsequent KDIGO guidelines in 2009 and 2017 did not specify ideal target levels of PTH for patients with CKD stages 3a to 5 who are not undergoing dialysis, but emphasise the significance of addressing modifiable factors in SHPT, such as hyperphosphataemia, hypocalcaemia or vitamin D deficiency, which can contribute to elevated PTH levels.^9^^,^ ^10^ For patients with stage 5 CKD undergoing dialysis, the guidelines advised maintaining PTH levels within two to nine times the URL of the assay.^9^^,^ ^10^ As a guideline from an Asian country, the 2012 Japanese Society for Dialysis Therapy (JSDT) guideline suggested a narrower target range of 60–240 pg/mL (6.6–26.4 pmol/L) for PTH levels in chronic dialysis patients.^12^ (Table 1)

Recent data revealed that lower PTH levels in patients with SHPT patients undergoing dialysis are associated with improved hypertension correction, reduced mortality and lower cardiovascular disease risk.^13^ However, achieving target PTH levels through medication alone can be challenging, necessitating invasive interventions. The 2009 and 2017 KDIGO guidelines recommend parathyroidectomy (PTX) for patients with CKD equal to or worse than stage 3a, with severe SHPT unresponsive to medication.^9^, ^10^ However, evidence shows that thermal ablation is an effective alternative treatment for refractory SHPT compatible with PTX.^14^ This review aims to comprehensively examine the treatment strategies of SHPT in patients with CKD. We particularly highlight comparisons among different therapeutic approaches regarding safety and effectiveness, providing clear understanding and guidance to the medical community for improving the quality of care for patients with CKD-associated SHPT.

## Therapeutic strategies for SHPT

**Fig. 1:**
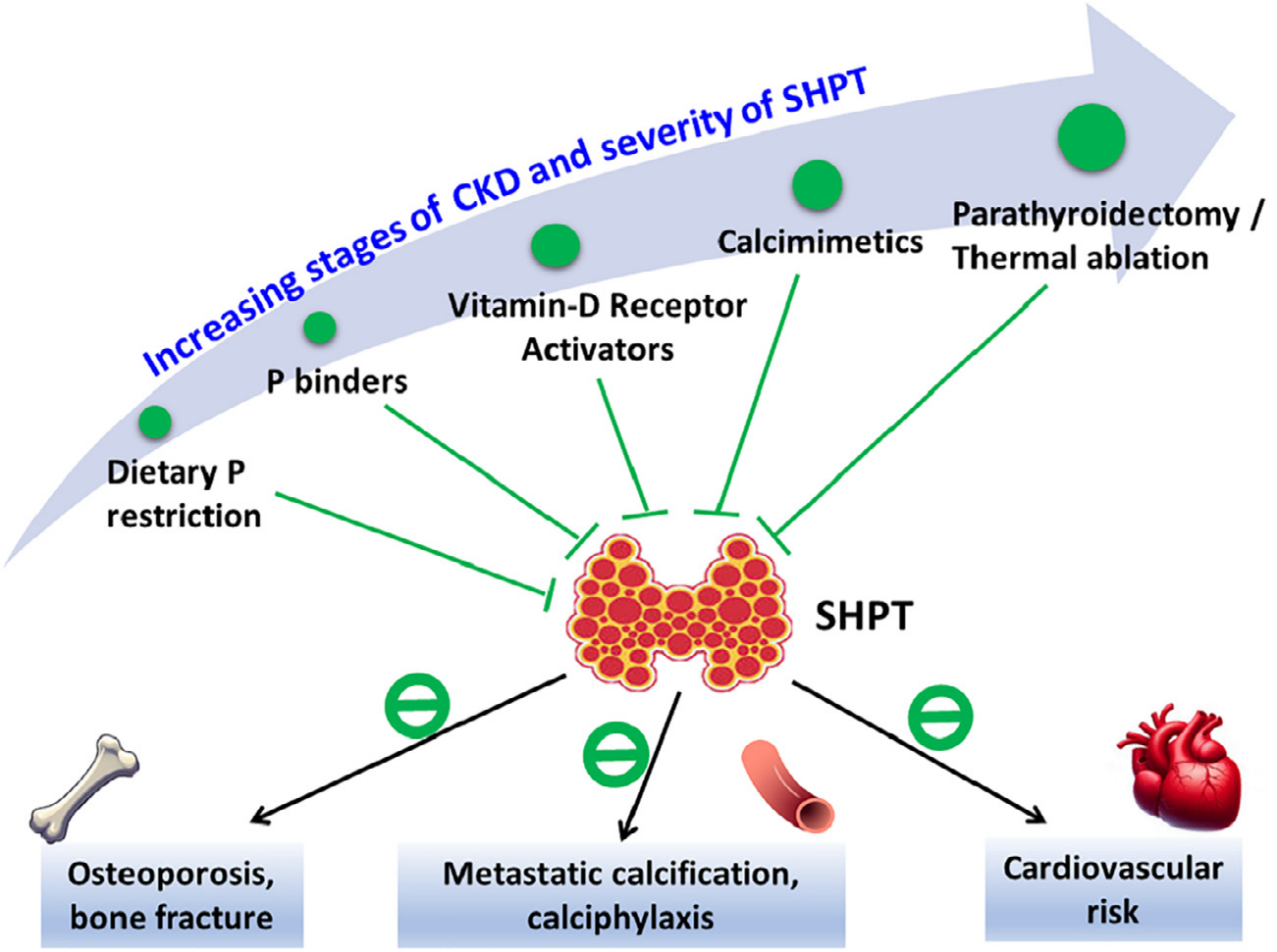
Overview of therapeutic options for SHPT. Note: The black lines denote the complications of SHPT, and the green lines denote the options for preventing or treating SHPT. Abbreviation: CKD, chronic kidney disease; P, phosphate; SHPT, secondary hyperparathyroidism.

With the objectives focusing on normalising serum phosphate, calcium and PTH levels, the therapeutic options are varied depending on the stages of CKD and severity of SHPT, to prevent subsequent complications of SHPT (Fig. 1).

The first step for the patients diagnosed with SHPT, regardless of the stages of CKD, is evaluation and correction of the modifiable factors including hyperphosphataemia, hypocalcaemia and vitamin D deficiency. Dietary phosphate restriction is an essential strategy for managing patients with SHPT (Fig 1 and Table 2). As for the medications for treating SHPH, phosphate binder is the first drug of choice, while vitamin D receptor activator (VDRA) should be reserved for patients with CKD stage 4–5 with severe and progressive SHPT.^10^ In patients with CKD stage 5 undergoing dialysis, various options are suggested to control PTH levels better. These options include VDRA, calcimimetics or combination therapy.^10^ Finally, PTX or thermal ablation should be considered for patients with refractory SHPT who fail to respond to medical therapies (Fig 1 and Table 2).

**Table 2:** Therapeutic options for SHPT.

Therapeutic options	Therapeutic target	Adverse effects and limitations of the therapy
Dietary phosphate restriction	Hyperphosphataemia	Increased risk of protein malnutrition
Phosphate binders	Hyperphosphataemia	
Calcium-based binders		Hypercalcaemia, soft tissue and vascular calcification, gastrointestinal upset
Calcium-free binders		
Aluminium-based binders		Aluminium toxicity
Iron-based binders		Diarrhoea, stool discoloration
Sevelamer/lanthanum		Gastrointestinal side effects

Vitamin D receptor activator	Target on vitamin D receptors, suppression of PTH synthesis	PTH level over suppression, hypercalcaemia

Calcimimetics	Target on calcium-sensing receptors, suppression of PTH synthesis	PTH level over suppression, hypocalcaemia

Parathyroidectomy	Parathyroid gland hypertrophy and hyperplasia	Transient (but possibly prolonged) hungry bone syndrome with hypocalcaemia, hoarseness due to surgery-related nerve damage, over-corrected PTH level

Thermal ablation	Parathyroid gland hypertrophy and hyperplasia	Lower response rate and higher recurrent rate compared to parathyroidectomy Less hypocalcaemia, hoarseness and hypoparathyroidism compared to parathyroidectomy

Abbreviations: PTH, parathyroid hormone.

### Medical treatment

#### Phosphate binder

Hyperphosphataemia is frequently observed in patients with advanced CKD and is an important factor contributing to SHPT development. Phosphate binder is an effective intervention for mitigating hyperphosphataemia, which attenuates the direct stimulatory effect on PTH secretion, reduces the formation of calcium–phosphate complexes responsible for hypocalcaemia-induced PTH secretion, and lowers FGF-23 levels, indirectly influencing PTH secretion.

Calcium-based phosphate binders, including calcium acetate and calcium carbonate, are commonly prescribed for dialysis patients and effectively reduce serum phosphate levels. However, their use may heighten the risk of vascular calcification and hypercalcaemia due to increased calcium phosphate precipitate formation. It is worth mentioning that the KDIGO 2017 guideline suggests restricting the dose of calcium-based phosphate binders to avoid calcium overload and subsequent metastatic calcification.^10^

On the other hand, calcium-free binders such as sevelamer, lanthanum and iron-based phosphate binders present a lower risk of vascular calcification. Nonetheless, evidence regarding their effects on bone and cardiovascular outcomes remains inconclusive.^15^^,^^16^ Some studies suggest that sevelamer and lanthanum may confer lower all-cause mortality than calcium-based phosphate binders, with fewer incidents of hypercalcaemia.^17^ However, iron-based binders may induce adverse effects such as diarrhoea.^18^

#### VDRA

In patients with CKD-induced SHPT, VDRAs mitigate the effects of vitamin D deficiency by binding to vitamin D receptors (VDRs) and inhibiting PTH synthesis through gene transcription regulation.^19^ VDRAs also improve hypocalcaemia by modulating genes involved in calcium channels and calcium-binding proteins, enhancing calcium balance.^20^^,^^21^ Additionally, VDRAs influence bone resorption and increase intestinal calcium absorption, raising serum calcium and phosphate levels, which further reduces PTH synthesis.^20^^,^^21^

VDRAs have limitations. Reduced expression of CaRs and VDRs in parathyroid gland hyperplasia may hinder PTH regulation by serum calcium and VDRAs.^22^ Despite this, VDRAs are valuable for managing SHPT in CKD. Calcitriol was the first VDRA for SHPT, while alfacalcidol, doxercalciferol and paricalcitol are effective prodrugs.^23^ Some studies suggest paricalcitol may offer better outcomes and survival than calcitriol.

Caution is needed as overuse of VDRAs, especially calcitriol can cause hypercalcemia and hyperphosphataemia, increasing vascular calcification risk.^24^ Newer VDRAs like paricalcitol are preferred for their moderate impact on hypercalcaemia,^25^ though evidence on cardiovascular benefits is limited.^26^

Emerging data support the early use of extended-release calcifediol (ERC) in CKD stages 3–4 due to high vitamin D deficiency. ERC provides steady calcitriol release, reducing immediate active vitamin D levels. Meta-analyses show similar PTH reductions to paricalcitol with fewer hypercalcaemia events. ERC may help prevent SHPT in early CKD.^27^

#### Calcimimetic

The calcium-sensing receptor (CaSR) is crucial as a receptor on parathyroid gland chief cells. In advanced CKD, hypocalcaemia and CaSR desensitisation due to gland hyperplasia increase PTH expression and secretion. Low extracellular calcium levels stabilise PTH mRNA, raising serum PTH levels.^28^ Calcimimetic agents mimic serum calcium's action on CaSRs, reducing PTH secretion. They also enhance CaSR expression and sensitivity.^29^ Clinical studies show that calcimimetics reduce parathyroid gland volume.^30^ Glands from treated patients had higher oxyphil/chief cell ratios^31^ and cystic degeneration,^32^ suggesting apoptosis as a treatment pathway for SHPT.

Calcimimetic agents have transformed CKD treatment, replacing phosphate binders and reducing vitamin D supplementation. In patients with SHPT, they improve biochemical outcomes, lowering serum PTH, calcium, phosphate and calcium–phosphate complex levels.^33^^,^^34^ Calcimimetics are linked to reduced PTX incidence, but evidence on mortality or cardiovascular risk is inconclusive.^35^ The Evaluation Of Cinacalcet HCl Therapy to Lower CardioVascular Events (EVOLVE) Trial showed fewer non-atherosclerotic cardiovascular events with cinacalcet, though not statistically significant.^36^

Commercially available calcimimetic agents include two oral forms (cinacalcet and evocalcet) and one intravenous form (etelcalcetide). Research by Palmer *et al* showed all three improved serum PTH levels short term. Etelcalcetide had the most significant reduction but a higher risk of hypocalcaemia, while cinacalcet caused nausea. Each agent has pros and cons, making preference challenging.^37^ A new injectable calcimimetic, Upacicalcet, which has only been licensed in Japan since 2021, is effective and safe for SHPT patients on dialysis.^38^ Combining cinacalcet with vitamin D significantly reduced serum phosphate, calcium–phosphate complex and PTH levels.^39^^,^^40^ The choice of agent depends on patient needs and tolerances. Evidence on the combination of calcimimetics and VDRAs regarding mortality and cardiovascular risks remains limited.

### PTX

#### Indication for PTX

Patients with SHPT often exhibit parathyroid hyperplasia, marked by the enlargement of the parathyroid glands. PTX entails tissue resection and can decrease serum PTH levels, thus ameliorating clinical symptoms. Studies have demonstrated that maintaining controlled serum PTH levels within the 21–150 pg/mL (2.3–16.5 pmol/L) range can significantly reduce all-cause mortality, yielding the most favourable survival outcomes.^41^ Hence, PTX may be warranted when patients demonstrate the following clinical manifestations despite prolonged medication usage (typically exceeding 6 months): (1) Exacerbation of clinical signs and symptoms, such as intensified bone, joint or muscle pain, systemic pruritus adversely affecting quality of life; (2) Onset of systemic complications, including the development of bone diseases like pathological fractures, calciphylaxis, symptomatic extra-skeletal manifestations, and deteriorating anaemia unresponsive to erythropoietin; (3) Progression of laboratory abnormalities, such as persistent hypercalcaemia or hyperphosphataemia despite medication adherence.^42^

The KDOQI guideline for CKD-MBD in 2003 defines severe hyperparathyroidism as persistently elevated serum intact-PTH levels exceeding 800 pg/mL (88 pmol/L), accompanied by hypercalcaemia or hyperphosphataemia, and exhibiting poor responsiveness to medical interventions.^43^ Subsequently, the KDIGO guidelines for CKD-MBD in 2009 and 2017 recommend PTX for patients with inadequate responses to medical or pharmacological treatment to enhance clinical outcomes.^9^^,^^10^

#### Comparisons among different approaches of PTX

There are three primary surgical approaches, namely total PTX (tPTX) with autotransplantation (AT), tPTX without AT, and subtotal PTX (sPTX).^42^ tPTX entails the identification and removal of all four parathyroid glands. In contrast, sPTX involves the removal of 3 to 3.5 parathyroid glands while leaving remnants at their original location. During surgery, transcervical thymectomy is often conducted to prevent recurrent SHPT, as ectopic or supernumerary parathyroid glands may persist within thymomas.^44^ In autotransplantation cases, the parathyroid tissue fragment is carefully assessed and transplanted, typically to the forearm's brachioradialis or the sternocleidomastoid muscle.

Roughly speaking, both tPTX and sPTX are safe and effective interventions for uncontrolled SHPT, that have demonstrated notable improvements in SHPT symptoms, including radiological enhancements in bone diseases, reduced time to recurrence, and decreased rates of persistent disease.^45^^,^^46^ These procedures also exhibit reduced cardiovascular risk and all-cause mortality compared to sole medical management.^47^ Regarding the outcomes comparisons between different surgery approaches, a systemic review and meta-analysis by Yuan *et al* found no significant differences in postoperative clinical and laboratory conditions between tPTX+AT and sPTX.^46^ On the contrary, a network meta-analysis by Hou *et al* revealed that postoperative hypocalcaemia occurs more frequently in patients with tPTX compared to sPTX or tPTX+AT, while no significant difference exists between sPTX and tPTX+AT. However, recurrence rates are significantly higher in sPTX than tPTX, tPTX+AT than tPTX, and sPTX than tPTX+AT. Additionally, patients with sPTX experience a higher reoperation rate compared to those with tPTX+AT or tPTX.^48^

Although inconclusive findings exist regarding the choices of surgery approaches, it is generally accepted that tPTX with AT emerges as the recommended optimal surgical approach for SHPT, offering maximal efficacy and safety with minimal adverse effects.^48^ Besides, tPTX is recommended for patients with a high risk of hypercalcaemia, particularly those with a history of prior neck surgery or laryngeal nerve injury or those considered high surgical risks.^49^ However, tPTX without AT is not advised for patients with planned renal transplantation. On the contrary, sPTX is preferable for patients at higher risk of hypocalcaemia, offering the benefits of reduced operation duration and shorter hospital stays.^45^^,^^46^

It is worth mentioning the essential postoperative complication, hungry bone syndrome (HBS), which causes acute, severe hypocalcaemia after PTX, typically occurring over 4 days post-surgery.^50^ HBS results from sudden PTH decline, halting bone resorption and leading to rapid mineralisation, calcium and phosphate uptake, and hypophosphataemia. Hypomagnesaemia in patients with CKD worsens hypocalcaemia. Studies highlight serum calcium, intact-PTH, phosphate and alkaline phosphatase (ALP) as crucial for identifying high-risk patients.^51^ Low preoperative calcium and elevated ALP/intact-PTH predict HBS.^52^^,^^53^

### Thermal ablation

For patients diagnosed with SHPT, invasive treatments are recommended if they exhibit symptoms or meet criteria such as persistent elevation in serum PTH levels, hypercalcaemia or hyperphosphataemia with inadequate medication response. However, thermal ablation offers an alternative for patients at high risk for general anaesthesia or those declining surgery. It provides rapid recovery, procedural simplicity and relative safety compared to PTX.^54^ Thermal ablation techniques used for SHPT include two most commonly utilised methods, microwave ablation (MWA) and radiofrequency ablation (RFA), and two less common methods, laser ablation and high-intensity focused ultrasound (HIFU). MWA operates within the electromagnetic spectrum, using frequencies of 915 MHz and 2.45 GHz. An antenna tip is inserted into the parathyroid gland, generating heat locally for tissue coagulation necrosis.^55^ RFA utilises energy from the spectrum between 3 Hz and 300 GHz. A needle guided by ultrasound creates an electrical circuit, delivering high energy to induce coagulation necrosis.^56^ Additionally, laser ablation delivers focused, high-energy light via an optical fibre, achieving efficient thermal ablation.^57^ HIFU converges ultrasound beams at a focal point, converting absorbed energy to heat and resulting in tissue necrosis.^58^

Research shows that MWA and RFA are effective and safe for treating SHPT. RFA significantly improves symptoms like restless legs, arthralgia, ostealgia, calcinosis cutis and pruritus. It also reduces serum PTH, calcium and phosphate levels immediately and 12 months post-treatment.^59^^,^^60^ A meta-analysis by Zhou *et al* in 2021, including 26 studies with 932 patients, found significant decreases in serum PTH at 1 and 6 months post-MWA. Reductions in serum calcium and phosphate at 6 months also indicated MWA's safety and effectiveness for SHPT.^54^ However, hypocalcaemia and transient hoarseness were common adverse events.^54^ Long-term follow-up showed significant PTH reduction over 60 months.^61^ A 2023 meta-analysis by Gong *et al* found that complete thermal ablation significantly reduced serum PTH, calcium and phosphate levels compared to partial ablation. There was no significant difference in severe hypocalcaemia incidence or symptom improvement between groups.^60^^,^^62^ Both MWA and RFA are effective for SHPT, with no significant differences in serum PTH, calcium and phosphate levels or adverse events up to 12 months post-ablation.^63^ MWA has a shorter operation time for single lesions and a higher complete ablation rate for lesions larger than 15 mm.^63^ Notably, thermal ablation therapy for SHPT is currently applied in a limited number of countries, mainly China, and is not yet widely utilised.

### Comparisons between calcimimetic and PTX

Despite PTX's potential advantages, its rates among end-stage renal disease patients with SHPT in the USA declined from 6.07 per 1,000 patients in 2004 to 3.67 per 1,000 in 2016, possibly due to increased cinacalcet use.^64^ Both cinacalcet and PTX improve serum calcium, phosphate and PTH levels, enhancing survival.^65^ However, PTX shows superior overall survival in dialysis patients, especially those with severe SHPT (intact PTH ≥500 pg/mL [55 pmol/L]).^65^^,^^66^ It significantly reduces serum calcium, phosphate and PTH levels in dialysis patients.^66^

Regarding cardiovascular risks, neither treatment reduces left ventricular mass, vessel, heart valve calcification or arterial stiffness.^66^ However, PTX is linked to fewer new cardiovascular events compared to cinacalcet.^67^ In patients with severe SHPT undergoing peritoneal dialysis, total PTX reduces osteopenia or osteoporosis incidence and increases bone mineral density in the lumbar spine and femoral neck.^68^

### Comparison between thermal ablation and PTX

Both thermal ablation and PTX are crucial for patients with refractory SHPT unresponsive to medication. A 2019 meta-analysis by Gong *et al*, involving six studies with 326 SHPT patients, compared the efficacy and complications of thermal ablation versus surgery.^69^ Results showed no significant differences in serum PTH, calcium or phosphate levels, or hoarseness incidence. However, thermal ablation reduced hypocalcaemia risk but increased persistent or recurrent SHPT risk.^69^

Recent cohort and randomised controlled trials comparing thermal ablation and PTX found that both methods achieved target serum PTH levels, though PTX often resulted in lower PTH levels.^70^, ^71^, ^72^, ^73^ No significant differences were observed in all-cause mortality or hoarseness; serum phosphate levels were similar. Some studies noted significant hypocalcaemia in the PTX group.^72^^,^^73^ RFA patients had similar success in reaching target PTH levels as PTX patients, with no differences in serum calcium and phosphate levels.^74^ RFA patients also had lower infection rates and shorter hospital stays. Preoperative bone-specific ALP concentration may predict postoperative hypocalcaemia. RFA was more cost-effective and may be an economical alternative. Thermal ablation is effective, with a lower hypocalcaemia risk and shorter hospital stays, making it a recommended option for recurrent SHPT patients (Table 3).

**Table 3:** Comparisons of thermal ablation and PTX in SHPT patients.

Treatment type	Study	Sample size (ablation/PTX)	Design	Dialysis duration (months)	Follow-up (months)	Study period	Baseline PTH level (ablation/PTX)	Target PTH level	Response rate	Adverse events
MWA vs PTX	Jiang *et al* (2019)	81 (33/48)	Cohort	10 vs 12	24	Jan 2015 to Dec 2017	1796/1460	124–558 pg/mL (13.6–61.4 pmol/L)	81.8% vs 52.6%	• No significance in hypocalcaemia and hoarseness

MWA vs tPTX	Wei *et al* (2020)	184 (92/92)	Cohort	96 vs 96	6	Mar 2018 to May 2019	1868/1746	-	-	• No significance in hypocalcaemia, Total PTX with longer recovery

MWA vs PTX	Diao *et al* (2021)	92 (47/45)	Cohort	8 vs 10	60	Jan 2010 to Mar 2019	1287/1446	150–600 pg/mL (16.5–66.0 pmol/L)	55.3% vs 31.1%	• Hypocalcaemia more in PTX • No significance in all-cause death

MWA vs PTX	Zhao *et al* (2021)	167 (79/88)	Non-RCT	7.9 vs 7.9	3	Jun 2018 to May 2020	1437/2099	> 80% reduction	85% vs 93%	• Significant hypocalcaemia in PTX compared to MWA

RFA vs. PTX	Ren *et al* (2022)	100 (47/53)	Cohort	7.9 vs 7.7	24	Jun 2014 to Dec 2020	1747/1857	≤ 300 pg/mL (33.0 pmol/L)	64.1% vs 82.1%	• No significance in all-cause mortality • The RFA group had a lower infection rate and length of hospital stay

Abbreviations: MWA, microwave ablation; RFA, radiofrequency ablation; PTX, parathyroidectomy; RCT, randomised controlled trial; PTH, parathyroid hormone.

## Conclusions

SHPT in CKD is a complex disorder caused by imbalances in calcium, phosphate and vitamin D metabolism. SHPT increases the risk of bone deformities, vascular calcification, cardiovascular events and mortality. Initial management focuses on correcting biochemical abnormalities through diet, phosphate binders, VDRA and calcimimetics. However, some patients require invasive treatments like PTX or thermal ablation. PTX, especially tPTX with AT, is highly effective for severe or refractory SHPT, offering better survival outcomes than medication alone. Thermal ablation techniques, such as microwave and radiofrequency ablation, are promising, less invasive alternatives, particularly for high-risk patients. Both PTX and thermal ablation effectively reduce SHPT-related complications. Future research should explore the molecular mechanisms of SHPT and the long-term efficacy of various treatments to improve patient care.

## Declaration of generative AI and AI-assisted technologies in the writing process

During the preparation of this work the authors used [Microsoft Bing / Copilot] for English editing. After using this tool/service, the authors reviewed and edited the content as needed and take full responsibility for the content of the publication.

## Funding

This review work received no external funding.

## Ethical statement

Not applicable for a review work.

## CRediT authorship contribution statement

**Shin-Hwa Tsai:** Writing – review & editing, Writing – original draft, Conceptualization. **Wei-Chih Kan:** Writing – review & editing, Writing – original draft, Conceptualization. **Rong-Na Jhen:** Writing – original draft. **Yu-Ming Chang:** Writing – original draft. **Jsun-Liang Kao:** Writing – original draft. **Hsien-Yung Lai:** Writing – review & editing, Writing – original draft, Conceptualization. **Hung-Hsiang Liou:** Writing – review & editing, Writing – original draft, Conceptualization. **Chih-Chung Shiao:** Writing – review & editing, Writing – original draft, Conceptualization.

## Declaration of competing interest

The authors declare that they have no known competing financial interests or personal relationships that could have appeared to influence the work reported in this paper.
